# Mitigating cyclophosphamide-associated gonadotoxicity in male *Wistar* rats: exploring the therapeutic potential of hesperidin

**DOI:** 10.3389/fvets.2024.1376225

**Published:** 2024-05-30

**Authors:** B. Hari Priya, B. Ramya, Swathi Bora, P. Shivakumar, A. Rohan, T. Vagdevi, A. Amoolya Rao

**Affiliations:** ^1^Department of Veterinary Pathology, College of Veterinary Science, Hyderabad, India; ^2^Department of Veterinary Pharmacology and Toxicology, College of Veterinary Science, Hyderabad, India; ^3^Intern, Deprtment of Internal Medicine, Mallareddy Institute of Medical Sciences, Hyderabad, India

**Keywords:** cyclophosphamide, hesperidin, *Wistar* rats, reproductive toxicity, oxidative stress, sperm parameters, NF-κB, inflammation

## Abstract

Hesperidin, a bioactive flavanone glycoside prevalent in citrus fruits, with remarkable therapeutic properties stands out as a formidable defender against the debilitating reproductive toxicity associated with Cyclophosphamide (CYP) chemotherapy. This study explores the protective potential of hesperidin (HSP@100 mg/kg b.wt PO daily) against CYP-induced (@ 40 mg/kg b.wt IP once in a week) reproductive toxicity in male *Wistar* rats as several studies were documented on single dose toxicity of CYP. In this experiment, we chose multidosage drug effects, which are more relevant in chemotherapy. Twenty-four rats were divided into four groups: Group 1 (Control), group 2 (CYP-treated), group 3 (HSP-treated), and group 4 (CYP + HSP-treated) for 28 days. The experimental design included assessments of relative testicular weight, semen analysis, testosterone levels, oxidative stress markers, inflammatory cytokines, gross and histopathological changes, and immunohistochemical evaluation. The results revealed that the administration of CYP led to a significant reduction in testicular weight, sperm count, motility, and testosterone levels, accompanied by increased oxidative stress and inflammatory response. Hesperidin co-administration demonstrated a protective effect by restoring these parameters to near-normal levels. Histopathological analysis revealed improved testicular architecture in the group 4 compared with the group 2. Oxidative stress indices indicated that hesperidin attenuated CYP-induced damage by reducing malondialdehyde levels, enhancing superoxide dismutase activity and maintaining glutathione levels. Similarly, inflammatory cytokine analysis demonstrated anti-inflammatory effects of hesperidin by reducing tumor necrosis factor-alpha (TNF-α) and elevating interleukin-10 (IL-10) levels in the group 4. Immunohistochemical evaluation of nuclear factor-kappa B (NF-κB) revealed increased inflammation in the CYP group, while hesperidin significantly reduced NF-κB expression, suggesting its anti-inflammatory properties.

## 1 Introduction

Worldwide, cancer ranks as a prominent factor leading to death in humans and canine population (1). It has been estimated that 15–30% of canine fatalities within a 3-year timeframe were attributed to cancer, as documented in the research conducted by Bronden (2). Similarly, 19.3 million new cases and over 10 million cancer-related deaths in 2020 were reported by the International Agency for Research on Cancer (IARC) (3). Apart from surgical procedures and radiation therapy, chemotherapy is a prevalent therapeutic approach used for animals suffering from cancer. Chemotherapeutic drugs, particularly cyclophosphamide (CYP) an alkylating nitrogen mustard, have significantly advanced cancer treatment as alone and in combination with other drugs but have severe side effects attributed to drug-induced oxidative and nitrosative stress. It is used clinically for the treatment of various neoplastic conditions such as lymphoma, myeloma, leukaemia, mycosis, neuroblastoma, adenocarcinoma, retinoblastoma, ovarian carcinoma, and breast carcinoma (4). It is also used as an immunosuppressor after organ transplantation and autoimmune diseases such as rheumatoid arthritis, myasthenia gravis, multiple sclerosis, lupus erythromatous, Wegener’s granulomatosis, and nephritic syndrome (5) also as a defleecing agent. CYP, derived from oxazaphorphorine, elicits therapeutic effects by providing alkyl groups that covalently bind with DNA. This prodrug undergoes hepatic microsomal cytochrome P450 activation to generate active metabolites. Initially converting into 4-hydroxycyclophosphamide, it reaches equilibrium with aldophosphamide. Aldophosphamide, entering target tissues, spontaneously transforms into phosphoramide mustard and acrolein. While phosphoramide mustard drives therapeutic activity as an alkylating agent, acrolein contributes to side effects, hypothesized to generate oxidative-free radicals such as hydroxyl, peroxides, and superoxide. This oxidative stress includes lipid peroxidation and glutathione depletion (6). Despite its therapeutic significance, the administration of elevated or multiple doses of cyclophosphamide has been associated with a diverse array of adverse effects. These include conditions such as hemorrhagic cystitis, alopecia, gonadotoxicity, and skin hyperpigmentation. Notably, reproductive toxicity has emerged as a prominent side effect observed in both human and experimental animal following cyclophosphamide treatment (7, 8). In the last few decades, diverse methodologies, incorporating antioxidant supplementation, have been investigated to ameliorate chemotherapy-induced stress. Clinical studies suggest that the synergistic application of chemotherapy and antioxidants may enhance patient survival rates.

Among the derivatives of botanical sources and dietary supplements, natural antioxidants exhibit potential to reduce drug-induced toxicity, thereby increasing the effectiveness of chemotherapy. Flavonoids are diverse group of phenolic compounds found abundantly in plants. They are key components of traditional remedies renowned for its efficacy (9). They work effectively as an antioxidant, anticarcinogen, and antiproliferative in countering multidrug resistance and in preventing chemotherapy-associated injury (10–13). Hesperidin, a bioflavonoid glycoside found in citrus fruits, demonstrates diverse therapeutic properties, including anti-inflammatory, antiapoptotic immunomodulatory, cardioprotective, and antioxidant effects (14, 15). Hesperidin mitigates inflammation by inhibiting the p38 MAPK signaling pathway, which is known for regulating key inflammatory mediators. This inhibition reduces the expression of IL-1β, interleukin-6, IL-8, IL-18, and TNF-α in both macrophages and murine models (16). Pre-treatment of keratinocytes with hesperidin significantly diminishes Nuclear Factor-κB and phosphorylated p38 MAPK levels upon oxidative stress exposure (17). These characteristics may contribute to potential protective effects in various tissues, including reproductive organs, against oxidative stress and inflammation, which are common contributors to reproductive toxicity (18). Our research aims to investigate how hesperidin mitigates reproductive toxicity induced by CYP.

## 2 Materials and methods

### 2.1 Chemical source

Cyclophosphamide was brought from Cadila Health Care Ltd., Hyderabad, under trade name (Endoxan-N) and Hesperidin – M/s. TCI (India) Pvt. Ltd. by Hychem laboratories, Hyderabad-500020.

### 2.2 Experimental design

A total of 24 male *Wistar* rats, weighing approximately 200–220 g, aged 6–8 weeks were procured from Jeeva Life Sciences, Hyderabad, 1757/PO/ReBiBt/S/14 CPCSEA, Hyderabad.

#### 2.2.1 Ethical approval

The experimental protocol was approved by the Institutional Animal Ethics Committee (IAEC), College of Veterinary Science, Rajendranagar, Hyderabad (1/25/C.V.Sc, Hyd. IAEC).

A total of 24 male albino *Wistar* rats were divided into 4 groups consisting of 6 animals each. The animals were maintained in polypropylene cages with a 12-h dark/light cycle at the animal house under hygienic conditions with an ambient temperature of 22–24°C and relative humidity of 30–70%. Rats were housed for 1 week for acclimatization before the start of the experiment. Throughout the experiment, the animals were fed with commercial standard and sterile feed in the form of pellets and provided with *ad libitum* potable drinking water.

Animals were under constant surveillance for any changes in behavior or health indicators. Physiological parameters such as weight, temperature, and hydration levels were monitored to ensure the well-being of the animals. Additionally, any deviations from normal activity levels or signs of distress were promptly addressed and documented.

Group I—Control.

Group II—CYP (@ 40 mg/kg b.wt IP once in a week) (19).

Group III—HSP (@ 100 mg/kg b.wt daily Peroral) (20).

**Figure 1:**
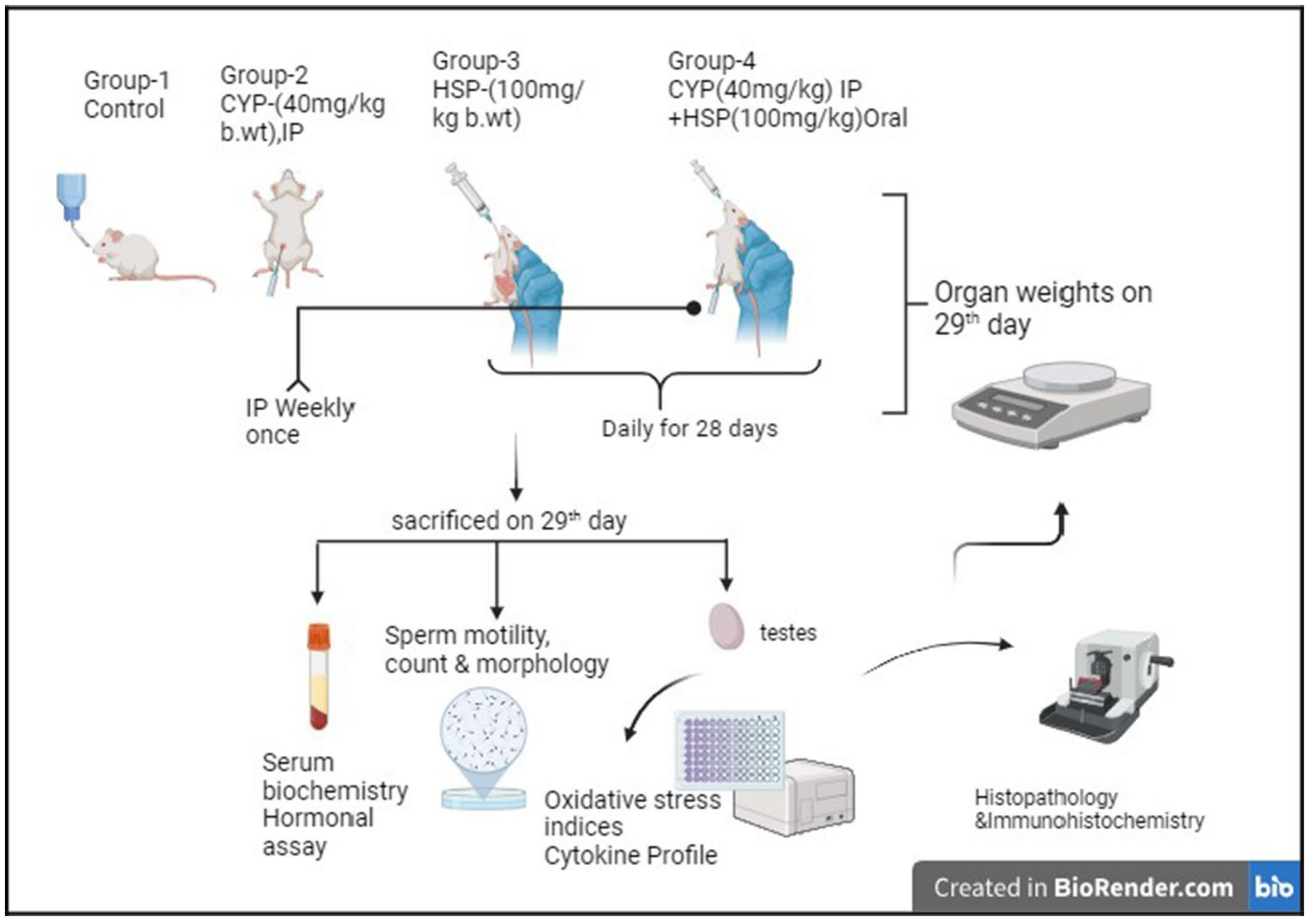
Experimental design.

Group IV—CYP + HSP (@40 mg/kg b.wt IP once in a week +100 mg/kg b.wt). The dose regimens were administered orally daily for 28 days (Figure 1).

### 2.3 Sampling

On day 29, before sacrifice, animals were weighed and testes were removed and washed. A section from the organ was stored in 10% formalin and used for histopathology and immunohistochemistry, and remaining parts were used for the estimation of markers of oxidative stress and inflammatory cytokine estimation.

### 2.4 Relative testicular weight

Rats were euthanized using CO2 chamber and underwent postmortem with gross observation of testes, and the tissue was collected, weighed, and stored at −80°C. Relative organ weight, expressed as a percentage of body weight, was calculated.

### 2.5 Semen analysis

Cauda epididymis was excised and macerated in warm saline to obtain epidydimal contents into a suspension and was used for semen analysis.

#### 2.5.1 Total sperm motility

Two drops of sperm suspension were taken on a sterile slide and cover slip was placed on it. Two slides were prepared in quick succession for each sample. The slides were examined under 40X, and the number of motile sperms was divided by the total number of spermatozoa counted and expressed as per cent motility (21).

#### 2.5.2 Total sperm count

An aliquot of the epididymal sperm suspension was utilized for spermatozoa count using Neubauer’s hemocytometer with a WBC diluting pipette. The epididymal fluid was drawn up to the “0.5” mark on the pipette, and the semen diluting fluid (consisting of 5 g sodium bicarbonate, 1 mL formalin, and 99 mL distilled water) was drawn up to the ‘11’ mark and these were mixed well. The hemocytometer chamber was charged, and the sperms were allowed to settle. The sperm count was conducted under a light microscope at 100X.

#### 2.5.3 Sperm morphology

To determine the sperm morphology, one drop of undiluted and liquefied semen was mixed with equal quantity (one drop) of Eosin–Nigrosin staining solution. This suspension was kept for 2-min incubation at room temperature. In total, 12 μL of this suspension was used to prepare smears by transferring a drop onto microscopic glass slide. After drying, the slides were examined for sperm morphology under light microscope at 100X (oil immersion) (22).

### 2.6 Testosterone analysis

The testosterone in the serum of all the rats was analyzed using Crystal Chem’s Mouse Testosterone ELISA Kit (catalog # 80552), which is an enzyme-linked immunosorbent assay, based on the principle of competitive binding. The test was performed according to the standard protocol provided by the manufacturer’s kit.

### 2.7 Oxidative stress indices

Small tissue samples were collected and stored at −20°C for analysis of organ antioxidant profiles, including glutathione (GSH), superoxide dismutase (SOD), and thiobarbituric acid reactive substances (TBARS). Tissue homogenization and subsequent centrifugation were performed to assess oxidative stress indices. Lipid peroxidation was evaluated by measuring malondialdehyde (MDA) using the TBARS method, according to the standard protocol (23). Tissue protein content was determined following the protocol outlined (24), while GSH concentration was measured using the procedure described by Moron et al. (25). SOD activity levels were determined according to the protocol provided by the study mentioned in Balasubramanian et al. (23) and Madesh and Balasubramanian (26).

### 2.8 Cytokine estimation

Specific inflammatory biomarker estimation was done by using enzyme-linked immune sorbent assay (ELISA) kit procured from Genelia, Krishgen Bio systems (Mumbai). ELISA plates are pre-coated with primary monoclonal antibodies. Rat lung tissue (100 mg) was homogenized with tris buffer and then centrifuged the homogenized sample, and the supernatant was taken and used for analysis. Wells were washed five times with wash buffer. Samples were added to microwells followed by standard with the same amount and incubated for 12 h at 4 ^0^c and then again washed with help of wash buffer two times, followed by adding 100 μL of detected antibody and incubated for 1 h. Biotin-labeled antibody is added followed by streptavidin-horseradish peroxidase (HRP) to wells and incubated for 20 min to form a complex and again washed with wash buffer and added the substrate solution to form (TMB) to microwells. Color absorbance is measured at 450 nm immediately after the color develops. Unit is measured in pg./mg protein.

### 2.9 Gross and histopathology

For histopathology examination, representative testicular samples were fixed in 10% neutral buffered formalin, then washed and dehydrated, processed in alcohol, cleared in xylol, and then embedded in paraffin. Paraffin blocks were sectioned into 5 μ slices, placed on glass slides, and stained with Hematoxylin and Eosin, and observations were made under light microscopy (27).

### 2.10 Immunohistochemistry

Immunohistochemistry was conducted to assess the immunoexpression of NF-κB in testes tissue sections. Tissue preparation involved deparaffinization, xylene clearing, and graded alcohol hydration. Antigen retrieval was achieved using TRIS EDTA and proteinase K. Sections were treated with PolyExcel hydrogen peroxide and blocked with 3% BSA. Primary antibodies (NF-κB) were applied at a 1:100 dilution in 3% BSA and incubated overnight at 4°C. Positive slides were subjected to 30-min incubation with universal polymer reagent, followed by 10-min visualization using DAB chromogen. Hematoxylin staining for 30 s acted as a counterstain, and immunoexpression intensity was observed under a light microscope at 100× magnification (28). Sections of rat liver were used as the control for immunohistochemical staining specificity.

### 2.11 Statistical data analysis and interpretation

Current studied data parameters obtained were exposed to one-way analysis of variance (ANOVA) applying statistical package for social sciences (SPSS) version 15.0 in each group data for analysis. Differences in mean values are tested with Duncan’s multiple comparison tests and compared between groups for significance level with consideration of statistically significance at *p* < 0.05. For graph representation, Tukey’s multiple comparison test was followed.

## 3 Results

### 3.1 Relative testicular weight

**Figure 2:**
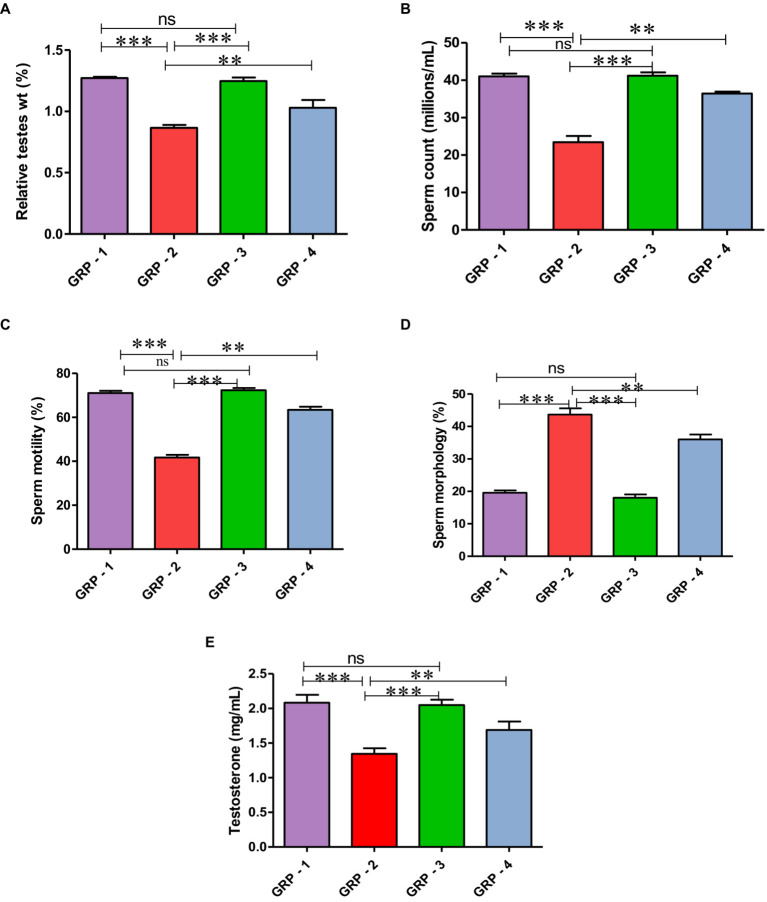
Relative testicular weights (%) in different groups **(A)**, mean values of sperm count (millions/mL) in different groups of rats **(B)**. Mean values of sperm motility (%) in different groups of rats **(C)**. Mean values of sperm morphology (%) in different groups of rats **(D)**. Mean values of testosterone (ng/ml) in different groups of rats **(E)**. Statistical analysis is done using one-way ANOVA to find differences between different groups (*p value, ***more significant difference, and **significant difference, ns—non significant difference), according to Tukey’s multiple comparison test.

The relative weights of testes were significantly (*p* < 0.05) lower in the group 2 rats when compared with group 1, group 3, and group 4. The mean values of groups 2 and 4 showed significant decrease in relative weights of testes when compared with groups 1 and 3. There was no significant difference in mean values between groups 1 and 3 (Figure 2A) (see Table 1).

**Table 1:** Semen analysis, oxidative stress and cytokine profiles of variuos groups.

Groups	Testes (%)	Testosterone (g/mL)	Sperm count (millions/mL)	Sperm motility (%)	Sperm morphology (%)	TBARS-μM of MDA/mg of tissue	GSH μM/mg protein	SOD-U/mg protein	TNF-α-pg/mL	IL-10-pg/mL
Group 1 (Control)	1.27 ± 0.01^a^	2.08 ± 0.11^a^	41.01 ± 0.76^a^	71.13 ± 0.97^a^	19.56 ± 0.74^c^	3.48 ± 0.20^c^	10.36 ± 0.24^a^	11.53 ± 0.38^a^	40.02 ± 0.92^c^	43.17 ± 1.54^a^
Group 2 (CYP)	0.86 ± 0.02^c^	1.34 ± 0.08^c^	23.45 ± 1.64^c^	41.66 ± 1.30^c^	43.66 ± 1.92^a^	7.23 ± 0.29^a^	8.11 ± 0.83^c^	8.18 ± 0.25^c^	64.33 ± 1.47^a^	21.57 ± 1.08^c^
Group 3 (HSP Control)	1.24 ± 0.10^a^	2.05 ± 0.07^a^	41.16 ± 0.94^a^	72.38 ± 0.97^a^	18.00 ± 1.06^c^	3.34 ± 0.18^c^	10.34 ± 0.29^a^	11.59 ± 0.36^a^	35.54 ± 2.09^c^	44.42 ± 1.46^a^
Group 4 (CYP+ HSP)	1.02 ± 0.06^b^	1.68 ± 0.12^b^	36.43 ± 0.49^b^	63.40 ± 1.42^b^	36.01 ± 1.52^b^	6.10 ± 0.37^b^	9.42 ± 0.17^b^	9.66 ± 0.27^b^	57.91 ± 1.18^b^	32.56 ± 1.07^b^

Values are Mean ± SE (*n* = 6); One-way ANOVA with Duncan’s post-hoc test (SPSS).

Means with different superscripts differ significantly (*p* < 0.05) among the groups.

### 3.2 Total sperm count, motility, and morphology

**Figure 3:**
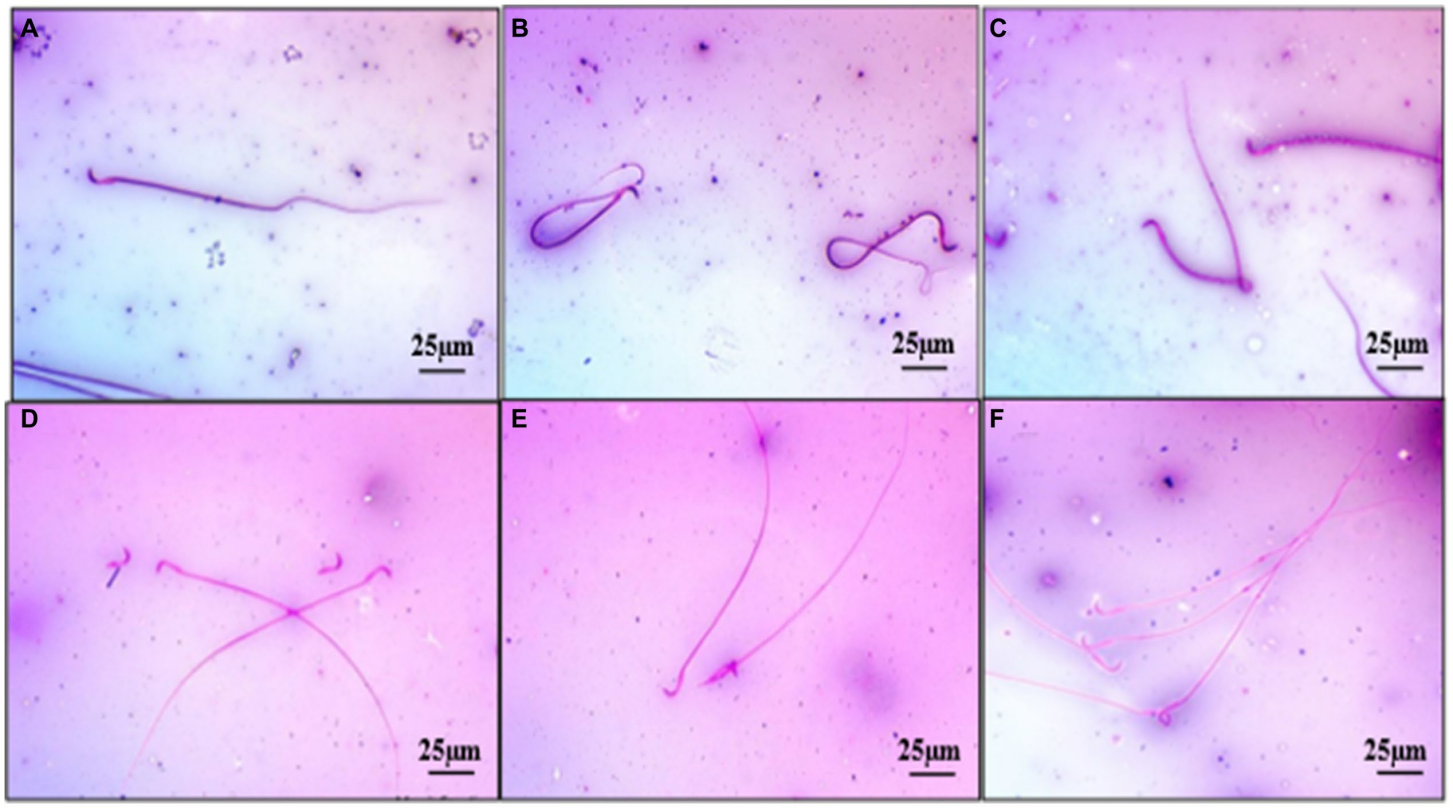
Photomicrograph showing normal spermatozoa with intact head (Group 1) **(A)**, abnormal sperm as hair pin loops (Group 4) **(B)**, sperm with bent midpiece (Group 2) **(C)**, sperms with detached heads (Group 2) **(D)**, sperms with broken neck (Group 2) **(E)**, sperm with distal cytoplasmic droplets (Group 4) **(F)** Eosin Nigrosine ×400.

A significant (*p* < 0.05) reduction was observed in the mean value of total sperm count (millions/mL) and motility (%) in the group 2 when compared with the group 1 rats. Treatment with ameliorative agent HSP showed a significant increase (*p* < 0.05) as compared with group 2. Similar concentration was observed in both groups 1 and 3 (Figure 2B). Sperm motility percentage (%) in all the groups was estimated. The mean values of the percentage of sperm motility (%) in the group 2 showed significant (*p* < 0.05) reduction in the number of motile sperms when compared with groups 1 and 3. Groups 1 and 3 showed similar values indicating safety of compound, while the group 4 showed a significant (*p* < 0.05) improvement as compared with the group 2 (Figure 2C). There was a significant (*p* < 0.05) increase in the mean value of abnormal sperm count in the group 2 compared with group 1, group 3, and group 4. Group 1 showed normal spermatozoa (Figure 3A). The abnormal sperm morphology includes sperms like hair pin loop (Figure 3B), bent mid piece (Figure 3C), detached heads (Figure 3D), and bent neck (Figure 3E). However, the treatment group 4 showed a significant (*p* < 0.05) decrease in the percentage of abnormal spermatozoa including mild degree of abnormality, showing distal cytoplasmic droplets (Figure 3F), as compared with group 2. There was no significant difference in the mean value of the group 3 compared with the group 1 (Figure 2D).

### 3.3 Testosterone

The serum testosterone (ng/mL) levels were significantly (*p* < 0.05) decreased in the group 2 when compared with group 1 and group 3. Serum testosterone levels in groups 1 and 3 were comparable, while group 4 showed significant (*p* < 0.05) increase as compared with the group 2 (Figure 2E).

### 3.4 Oxidative stress indices

**Figure 4:**
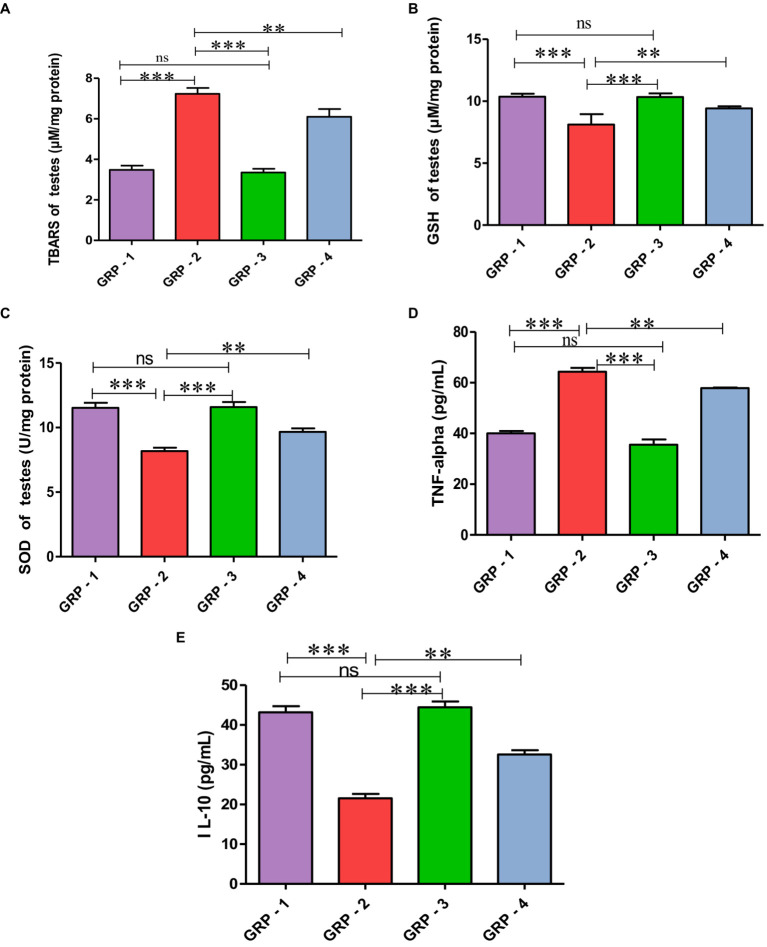
Protective effect of HSP on oxidative stress parameters TBARS **(A)**, GSH **(B)**, SOD **(C)**, HSP ameliorates CYP-induced testicular toxicity by significantly decreasing the pro-inflammatory cytokine profile TNF-α **(D)** and significantly increasing anti-inflammatory cytokines (pg/mL) IL-10 **(E)**. Statistical analysis is done using one-way ANOVA to find difference between different groups (*p value, ***more significant difference, and **significant difference, ns—non significant difference), according to Tukey’s multiple comparison test.

The oxidative stress markers in the testes were evaluated through measurements of malondialdehyde (MDA), glutathione (GSH) concentration, and superoxide dismutase (SOD) activity in different groups. In this experiment, group 2 experienced heightened oxidative stress, as evidenced by increased MDA levels, decreased GSH concentration, and reduced SOD activity. However, the group 4 demonstrated a protective effect, with improvements in all three parameters, compared with the group 2, indicating a potential therapeutic or preventive intervention against oxidative damage. Group 3, although not significantly different from the group 1, also exhibited trends toward antioxidant protection (Figures 4A–C).

### 3.5 Cytokine estimation

Evaluating inflammatory markers, the concentration of TNF-α in the group 2 was significantly elevated, and reduced concentration of IL-10 was observed compared with the group 1, indicating increased inflammation. Group 3, while not significantly different from the group 1, also demonstrated trends toward maintaining a balanced inflammatory profile. Intervention in the group 4 may contribute to a reduction in inflammation as evidenced by significant decreased TNF-α levels and increased in IL-10 levels compared with the group 2 rats. These findings highlight the potential anti-inflammatory effects of hesperidin, particularly attenuating the pro-inflammatory marker TNF-α and enhancing the anti-inflammatory marker IL-10 (Figure 4D).

### 3.6 Gross and histopathology (HP)

**Figure 5:**
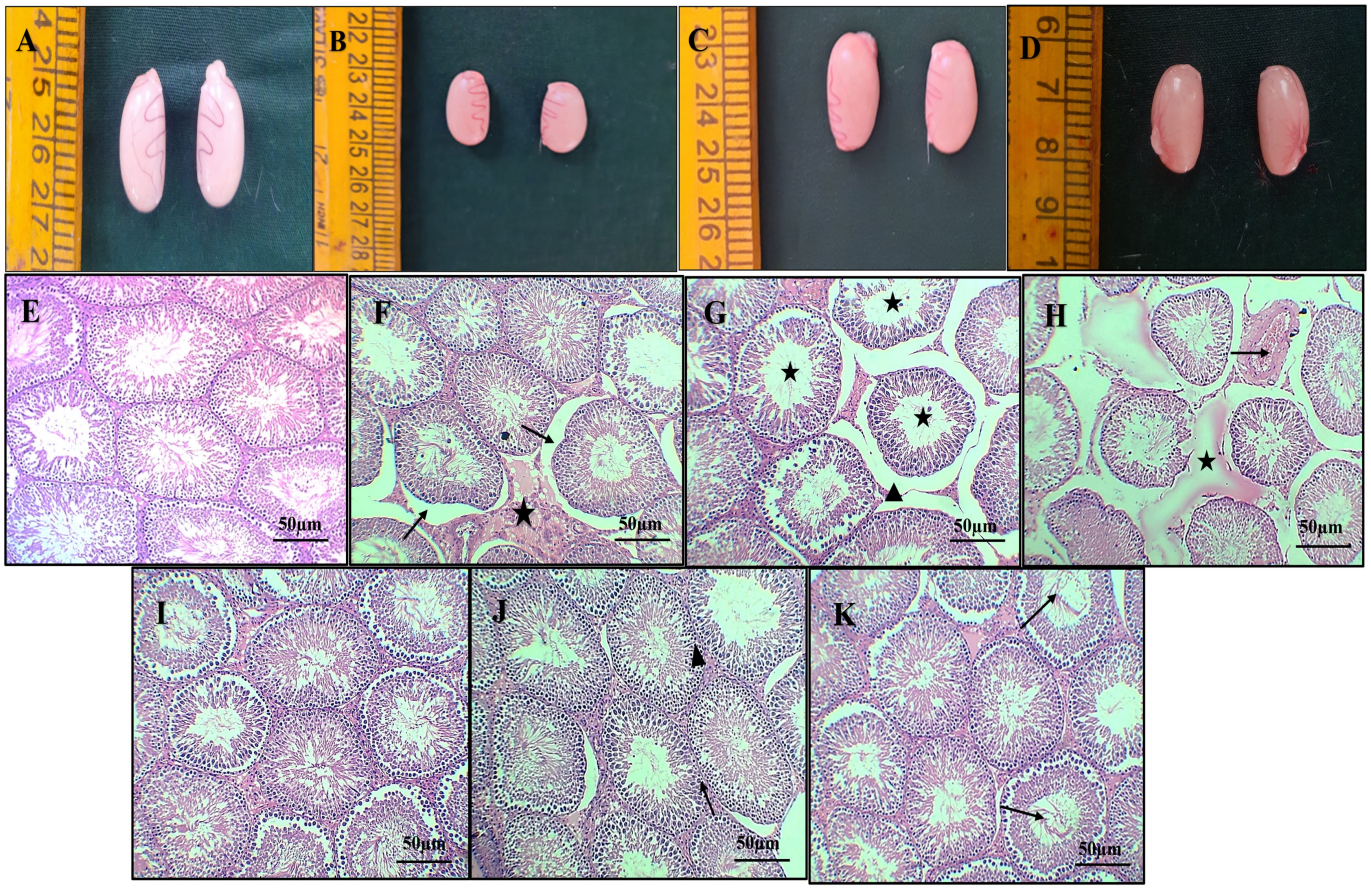
Gross pathology of the testes from different groups, normal appearance of testes [Group 1-**(A)**], severe congestion and mild reduction in size of the testes [Group 2-**(B)**], normal appearance of the testes [Group 3-**(C)**], showing mild congestion of the testes [Group 4-**(D)**]. Photomicrograph of histopathology of the testes (H & E X 10): normal architecture of seminiferous tubules with intact interstitial space [Group 1-**(E)**] [Group 3-**(I)**], moderate interstitial edema (star), desquamation of germinal epithelium from basement membrane (arrow) **(F)**, dilatation of intertubular space (arrow head) and hypo spermatogenesis (star) **(G)**, marked congestion of testicular blood vessel (arrow) and interstitial edema (star) **(H)** (Group 2). Intact germinal epithelium of seminiferous tubules (arrow) **(J)** and normal intertubular space (arrow head), restoration of spermatogenesis (arrow), and mild edema **(K)** (Group 4).

Macroscopically, the testes in groups 1 and 3 exhibited a normal and healthy appearance. In contrast, the group 2 displayed severe congestion along with a slight reduction in testes size. On the other hand, the group 4 showed mild testes congestion, indicating a milder impact compared with the group 2 (Figures 5A–D).

Histopathological sections of the testes from group 1 (Figure 5E) and group 3 (Figure 5I) showed normal architecture of seminiferous tubules with active spermatogenesis and normal interstitial space. Sections of group 2 rats revealed specific changes such as desquamation of germinal epithelium from basement membrane, marked hypospermatogenesis, moderate interstitial edema, spermatogenesis, moderate disintegrated seminiferous tubular epithelium, loss of germinal epithelium, congestion of testicular blood vessel, and intertubular space (Figures 5F–H). Sections from group 4 rats revealed intact germinal epithelium of seminiferous tubules, restoration of spermatogenesis and mild inter tubular edema, normal intertubular space, and active spermatogenesis (Figures 5J–K).

### 3.7 Immunohistochemistry

**Figure 6:**
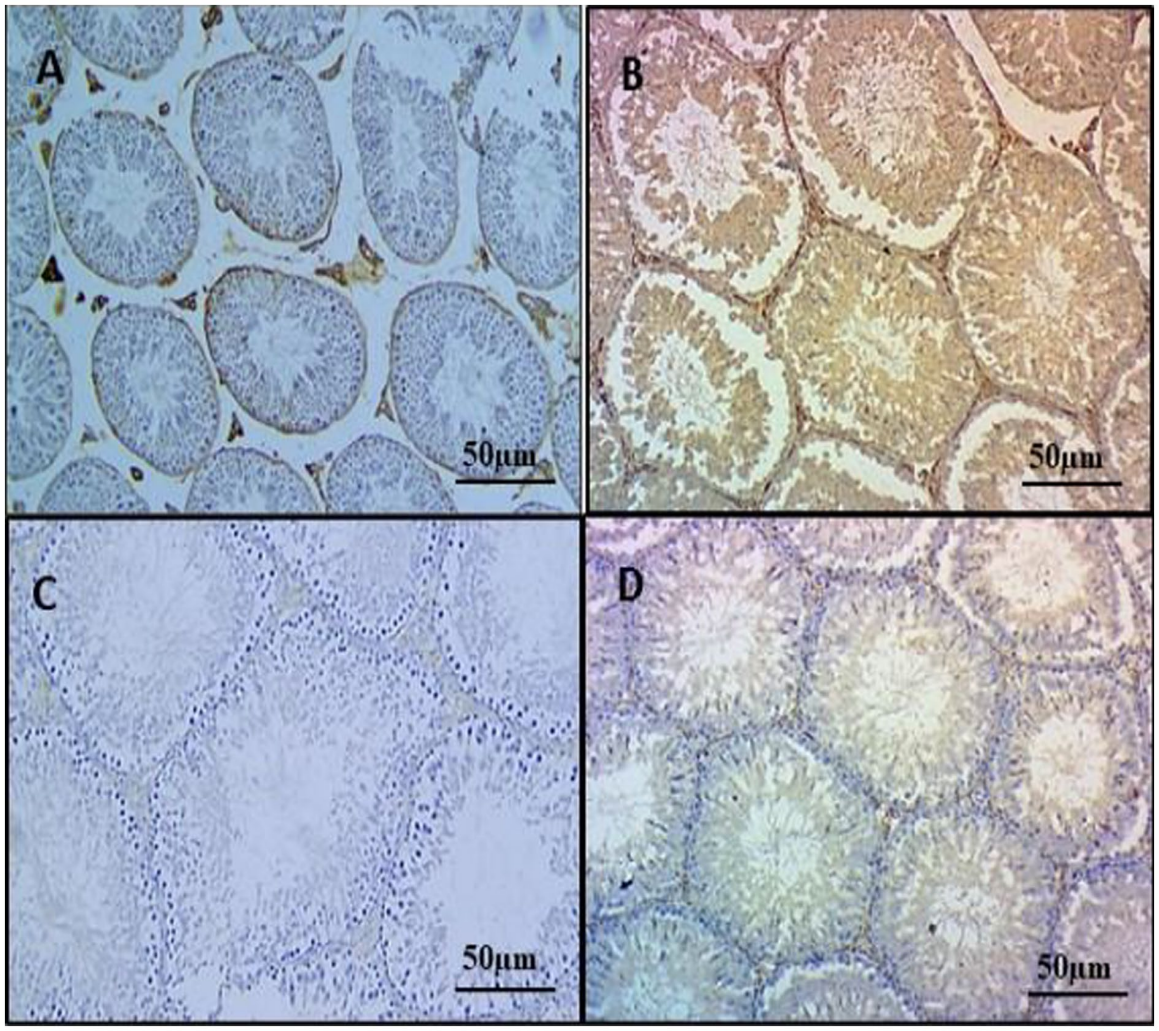
Effect of HSP on immunohistochemistry on testicular tissues IHC X 100. Testes showing very faint immunostaining for NF-κB in the testes (Group 1) **(A)**; testes showing strong immunoreactivity for NF-κB, positive staining cells include both seminiferous tubules and Leydig cells of the testes (Group 2) **(B)**; testes showing mild immunoreactivity for NF-κB in Leydig cells of the testes (Group 3) **(C)**; testes showing diminished immunoreactivity for NF-κB, no staining is appreciable in Leydig cells of the testes (Group 4) **(D)**.

To elucidate the protective mechanisms against inflammatory responses attributed to HSP, we conducted immunohistochemistry (IHC). Increased immunoreactivity of NF-κB was exhibited in testicular sections of group 2 rats due to enhanced staining intensity in positive cells, including seminiferous tubule, spermatogonia, and Leydig cells, markedly higher than groups 1, 3, and 4. Groups 1 and 3 displayed either no or low NF-κB immunoreactivity in testicular sections, while treatment with hesperidin in the group 4 exhibited significant diminished NF-κB activity in the testis tissue when compared with the group 2 alone (Figures 6A–D).

## 4 Discussion

The increasing prominence of gonadotoxicity stands out as a prevalent and frequently encountered consequence of prolonged CYP utilization. Consequently, there exists a pressing and vital requirement to explore therapeutic approaches aimed at mitigating or controlling the reproductive impacts faced by cancer patients undergoing CYP treatment. Several studies strongly suggest that antioxidant activity from natural compounds has a protective effect against chemical-induced toxicity of cyclophosphamide (29). The current study is conducted to assess the safeguarding effects of hesperidin against reproductive toxicity induced by cyclophosphamide. In the present study, group 2 rats showing decreased relative weights of the testes are due to a diminished number of germ cells, atrophy, and hypo spermatogenesis. Moreover, several studies support that the diminished presence of androgens could play a role in the reduction of organ weights (30), which correlates with the decrease in testosterone levels in the group 2 when compared with groups 1 and 3. This alteration might be due to enzymatic and non-enzymatic destruction of epididymal tissue by CYP (31). The concurrent administration of hesperidin resulted in the reinstatement of testosterone levels and the restoration of testes and epididymis in the group 4 (32).

A significant reduction in sperm count and motility is likely attributed to the impact of CYP in group 2 rats. Additionally, morphological analysis revealed a high percentage of coiled and looped tails, detached heads, and degenerated sperms in the same group. These changes are in alignment with previous studies (33, 34). Alterations in theses parameters show the multifaceted impact of cyclophosphamide on various aspects of sperm function and quality by inducing lipid and protein peroxidation, oxidative stress, and DNA damage along with decreased ATP levels, discreetly hindering sperm motility. These alterations in sperm morphology may be linked to impaired gonadotropin secretion and testosterone production, leading to a reduction in spermatogenic cells and adversely affecting sperm quality (35). Hesperidin co-administration significantly enhanced sperm count, motility, and morphology in group 4 rats, mitigating the adverse effects of oxidants, such as CYP (36).

Oxidative stress emerges as a pivotal factor in the progression of tissue damage, which is characterized by a disruption in the equilibrium between the production of reactive oxygen species (ROS) and capacity of the body to counteract or mend their detrimental impacts. Prolonged exposure to oxidative stress can significantly contribute to organ toxicity across various bodily systems. Therefore, the effective management of oxidative stress and reinforcement of antioxidant defenses stand as indispensable strategies in both averting and ameliorating tissue damage (37–39). In the present study, the group 2 exhibited a notable increase in malondialdehyde (MDA) levels, which is concurrent with a significant decrease in superoxide dismutase (SOD) and glutathione (GSH) compared with the other groups. These alterations emphasize the oxidative stress induced by cyclophosphamide (CYP) metabolism. The breakdown of CYP metabolites leads to the generation of reactive oxygen species (ROS), particularly disrupting the inflammatory pathway and causing an elevation in MDA levels. The heightened ROS levels further diminished the activities of SOD and depleted GSH levels. These excessive free radicals interact with polyunsaturated fatty acids in the phospholipids of cell membranes, inducing lipid peroxidation (LPO) in testis tissues. This collective impact reinforces the intricate relationship between CYP-induced oxidative stress and the observed changes in MDA, SOD, and GSH levels, underscoring the potential tissue damage associated with CYP administration (40, 41). In the group 4, hesperidin demonstrated an improvement in parameters including reduced malondialdehyde (MDA) levels, elevated superoxide dismutase (SOD), and glutathione (GSH). The role of hesperidin in neutralizing reactive oxygen species (ROS) serves as a vital defense against intrinsic and extrinsic elements, such as oncogenes and toxic compounds. By scavenging radicals, hesperidin restores the activities of SOD, catalase (CAT), and glutathione peroxidase (GPx), exhibiting potent antioxidant properties. Furthermore, hesperidin inhibits ROS-induced oxidative damage and apoptosis, contributing to the observed improvement in tissue conditions in the group 4 (42, 43).

In the group 2, a significant elevation in TNF-α levels was observed, correlating with oxidative stress-induced pro-inflammatory cytokine production and apoptosis. This increase is likely attributed to acrolein-mediated release of inflammatory cytokines by the activation of the nuclear factor kappa B (NF-κB), which further led to the production of cytokines as TNF-α and IL-6 in addition to ROS (44–46). Simultaneously, a notable reduction in IL-10 levels was observed in the group 2 compared with other groups, suggesting impaired upregulation due to severe oxidative stress injury. These findings emphasize the intricate interplay between oxidative stress, inflammatory cytokines, and the potential regulatory role of antioxidants (47, 48).

Hesperidin demonstrated significant anti-inflammatory effects in the group 4, as evidenced by a notable decrease in TNF-α concentration compared with the group 2 (*p* < 0.05). This reduction is linked to the ability of hesperidin to suppress ERK phosphorylation, thereby inhibiting the activation of inflammatory genes. Additionally, the group 4 exhibited a significant increase in IL-10 concentration (*p* < 0.05) compared with the group 2. The elevated IL-10 levels can be attributed to the role of hesperidin in reducing the nuclear translocation of NF-κB. Overall, these findings highlight the potential of hesperidin in mitigating inflammation and reinforcing antioxidant defenses in the group 4 (49).

The susceptibility of testicular tissues to oxidative damage arises from the presence of ROS-generating systems, including xanthine oxidase, NADPH oxidase, and the mitochondrial electron transport chain, which are compounded by high concentrations of PUFA. Given that oxidative damage significantly contributes to testicular dysfunction, safeguarding these tissues is crucial (50). In group 2 rats, histological examination revealed a compromised testicular architecture, with widened interstitial space, edema, degeneration, and desquamation of germinal epithelium, and seminiferous tubules. The decreased number of spermatids, spermatocytes, and oxidative stress indices indicates free radical-induced damage to the testes. These degenerative alterations likely impede spermatogenesis, leading to reduced sperm concentration and the production of abnormal sperm, ultimately affecting male fertility (30, 31, 51). In contrast to the group 2, the testicular sections of the group 4 exhibited milder microscopic changes, indicating a potential protective influence of hesperidin treatment. The observed benefits are likely attributed to the antioxidant activity of hesperidin, as evidenced by a significant reduction in testicular MDA levels. This reduction correlates with the restoration of the seminiferous tubular pattern and enhanced proliferative activities of spermatogonial cells. Overall, these findings suggest that antioxidant properties of hesperidin contribute to the amelioration of testicular damage and support spermatogenesis in the group 4 (30, 32).

In this study, the group 2 exhibited strong positive immunoreactivity, indicating inflammation. NF-κB, a redox-sensitive transcription factor, regulates pro-inflammatory cytokines such as IL-6, TNF-α, and iNOS. The increased NF-κB staining intensity in the group 2 suggests CYP-induced inflammation, which is supported by elevated TNF-α levels (52, 53). Notably, the group 4 showed a significant reduction in NF-κB expression, potentially attributed to the ability of hesperidin to inhibit NF-κB activity, suppress IkB phosphorylation, and decrease reactive oxygen species, highlighting its anti-inflammatory properties (54). These similar ameliorative actions of HSP were observed in the study mentioned in the Rezaei et al. (52) and Abd El Tawab et al. (53).

**Figure 7:**
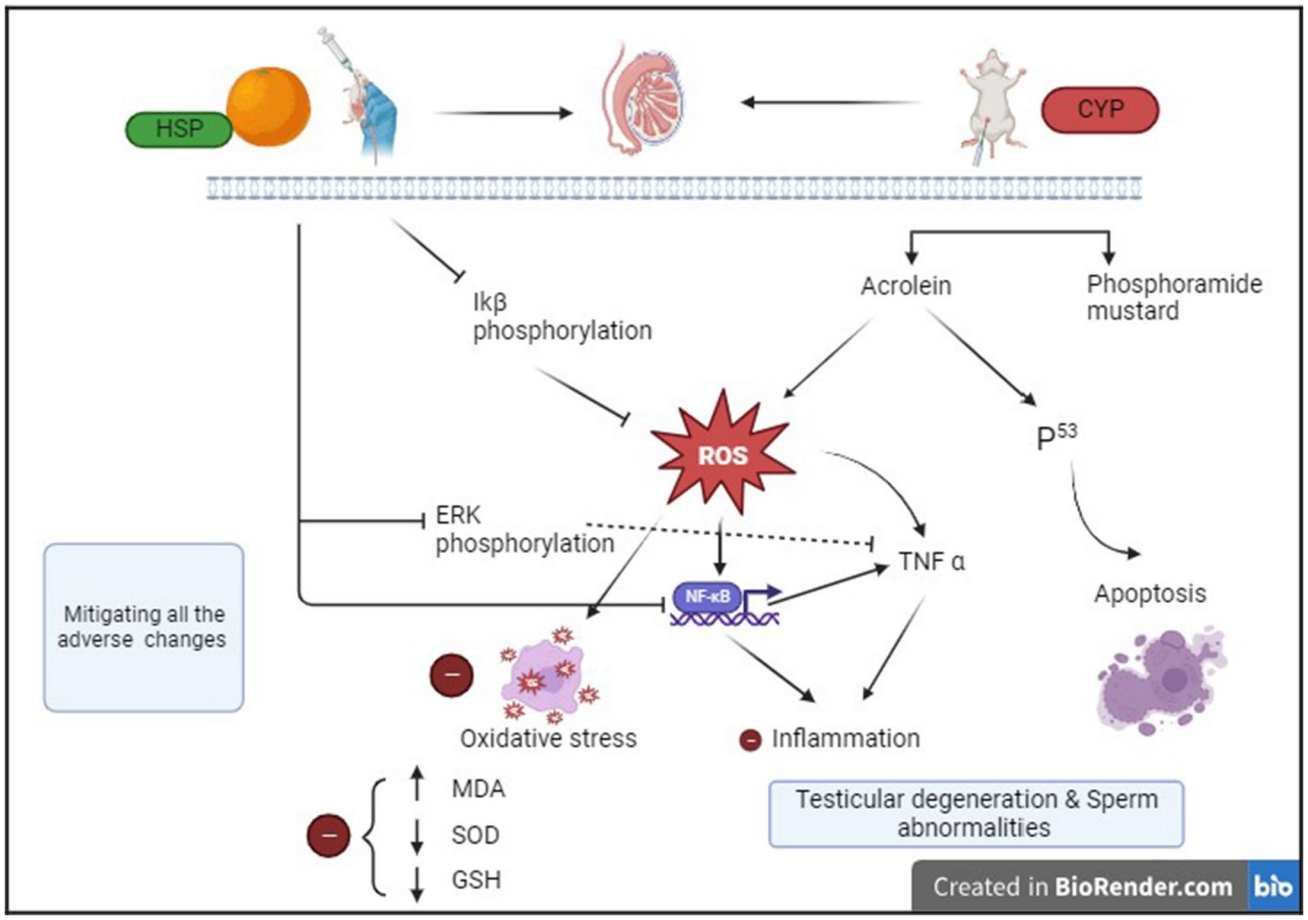
Schematic diagram of ameliorative effect of HSP on CYP-induced testicular toxicity.

Hence, our findings speculated that HSP has strong protective action of antioxidant by scavenging ROS and anti-inflammatory properties, suppressing NF-κB signaling pathway. This possible mechanism of action is shown in Figure 7.

## 5 Conclusion

The study concludes that the gonadotoxic effects induced by multidose injections of CYP are mitigated by HSP. The observed pathological changes, resulting from the administration of CYP, are ameliorated by HSP, showcasing its multifaceted protective mechanisms. These effects are attributed to the antioxidant, anti-inflammatory, and antiapoptotic properties of HSP, suggesting its potential as a therapeutic intervention against CYP-induced gonadotoxicity.

## 6 Limitations of the study

The findings presented in this research give valuable information regarding the protective role of hesperidin in cyclophosphamide-induced reproductive toxicity in male *Wistar* rats. Nevertheless, as with any scientific inquiry, there are some constraints to consider when interpreting the findings:

*Model specificities of animals:* The test model employed was male *Wistar* rats. Although it is usual to study toxicology through these animals due to their physiological similarity with humans, its direct application to human population demands utmost caution. There can be significant differences between humans and rats in terms of human physiology and drug reactions, which may affect generalizability of the latter.

*Mechanistic understanding:* The sample size used in this study is relatively small, consisting of six animals per group. While this number may be useful for detecting large effect sizes, it might have limited statistical power to detect smaller intergroup differences.

*Single compound intervention:* The study focuses solely on the protective effects of hesperidin against cyclophosphamide-induced toxicity. While hesperidin has demonstrated antioxidant and anti-inflammatory properties in various studies, the use of a single compound intervention may overlook potential synergistic effects that could arise from the combination of hesperidin and other antioxidants or therapeutic agents.

*Translation and clinical practice:* While the findings of this study are promising in a preclinical animal model, translating these results to clinical practice requires further validation through clinical trials in human populations. Factors such as bioavailability, pharmacokinetics, and potential interactions with other medications need to be considered in the translation process.

## 7 Background

Hesperidin plays pivotal role in scavenging ROS, counteracting inflammation and enhancing reproductive parameters. Recent studies have focused on hesperidin supplementation to address ovarian toxicity-induced oxidative stress and infertility in animal models, showcasing promising results such as improved follicular development and hormonal balance, and this made hesperidin an attractive target for therapeutic interventions of reproductive studies. Although research on hesperidin's impact on male reproductive toxicity is limited, its potential to improve reproductive parameters justifies further investigation. This underscores the rationale for considering hesperidin as a therapeutic agent for mitigating male reproductive toxicity.

## Data availability statement

The original contributions presented in the study are included in the article/supplementary material, further inquiries can be directed to the corresponding author.

## Ethics statement

The experimental protocol was approved by the Institutional Animal Ethics Committee (IAEC), College of Veterinary Science, Rajendranagar, Hyderabad (1/25/C.V.Sc, Hyd. IAEC).

## Author contributions

BH: Conceptualization, Formal analysis, Funding acquisition, Validation, Writing – original draft. BR: Conceptualization, Formal analysis, Supervision, Writing – original draft. SB: Conceptualization, Formal analysis, Supervision, Writing – original draft. PS: Conceptualization, Supervision, Writing – original draft. AR: Writing – review & editing. TV: Writing – review & editing. AA: Data curation, Formal analysis, Investigation, Methodology, Resources, Software, Writing – review & editing.
